# Investigation gene and microRNA expression in glioblastoma

**DOI:** 10.1186/1471-2164-11-S3-S16

**Published:** 2010-12-01

**Authors:** Hua Dong, Hoicheong Siu, Li Luo, Xiangzhong Fang, Li Jin, Momiao Xiong

**Affiliations:** 1 State Key Laboratory of Genetic Engineering and MOE Key Laboratory of Contemporary Anthropology, School of Life Sciences and Institutes of Biomedical Sciences, Fudan University, Shanghai, 200433, China; 2 Human Genetics Center, University of Texas School of Public Health, Houston, TX 77030, USA; 3 School of mathematical sciences, Peking University, Beijing, 100871, China

## Abstract

**Background:**

Glioblastoma is the most common primary brain tumor in adults. Though a lot of research has been focused on this disease, the causes and pathogenesis of glioblastoma have not been indentified clearly.

**Results:**

We indentified 1,236 significantly differentially expressed genes, and 30 pathways enriched in the set of differentially expressed genes among 243 tumor and 11 normal samples. We also indentified 97 differentially expressed microRNAs among 240 tumor and 10 normal samples. 22 of which have been reported to affect glioblastoma and 50 of which were implicated in other cancers and brain diseases. We regressed gene expression on microRNA expression in 237 tumor tissues and 10 normal tissues comprehensively. We found two experimentally validated microRNA targets and 1,094 miRNA-target gene pairs in our datasets which were predicted by miRanda algorithm, 8 of the target genes were tumor suppressor genes and 3 were oncogenes. Further function analysis of target genes suggested that microRNAs most frequently targeted genes associated with Cell Signalling and Nervous System.

**Conclusion:**

We investigated gene and microRNA Expression in Glioblastoma and gave a comprehensive function study of differential expressed gene and microRNA in glioblastoma patients. These findings gave important clues to study of the carcinogenic process in glioblastomas.

## Background

Glioblastoma Multiforme (GBM) is the most common and most aggressive type of primary brain tumor, accounting for 52% of all primary brain tumor cases and 20% of all intracranial tumors [1]. Primary GBM arise de novo, without any history of pre-existing lower-grade tumor, whereas secondary GBM have clinical, radiologic, or histopathologic evidence of malignant progression from pre-existing lower-grade tumor [2]. In the past two decades, the molecular mechanisms, genetics and paths to treatment of Glioblastoma have extensively been studied [3]. However, the causes and pathogenesis of glioblastoma have not been indentified clearly. With the continuing improvement of high-throughput genomic technologies, it is now feasible to survey human cancer genomes comprehensively. The Cancer Genome Atlas (TCGA) aims to catalogue and discover major cancer-causing genome alterations in large cohorts of human tumors through integrated multi-dimensional analyses [4]. Glioblastoma is the first cancer studied by TCGA. To identify the genetic alterations in glioblastoma, we investigated the expression profiles of gene and microRNA.

MicroRNAs (miRNAs) are single-stranded short coding RNA molecules of about 22 nucleotides in length, which usually repress gene expression by binding at the 3’UTR region of target gene [5]. The expressions of microRNAs are found to be highly different in organ development and tissue differentiation [6]. Moreover, many microRNAs have been found to associate with apoptosis and cancer, suggesting they function as oncogene or tumor suppressor gene [7]. In our study, we examined the expression levels of 470 human miRNAs in glioblastoma and indentify a group of microRNAs whose expression is significantly altered in this tumor. We also indentified the significantly altered gene expression and pathways related to glioblastoma.

## Results

All types of data were acquired from TCGA project [4] (http://cancergenome.nih.gov/dataportal/data/about/). Gene expression microarrays were performed on Affymetrix HT Human Genome U133 Array Plate Set by Massachusetts Institute of Technology (MIT). Level three data gave calls for genes per sample after Probeset-level and Gene-level Robust Multiarray Analysis (quantile normalization and background corrected) until the most recent update on Sep. 05, 2008. After calculation the average expression values for duplicated samples, finally 243 tumor tissue samples, 10 normal tissues and 1 cell line sample from glioblastomas patients were used for differential expression analysis. MicroRNA expression experiments were performed on Agilent 8 x 15KHuman microRNA-specific microarray by Universities of North Carolina (UNC). There are 534 microRNAs (470 human microRNAs) and 240 tumor tissue samples, 10 normal tissue samples available in level three data (after quantile normalization and batch adjusted) until the most recent update on Nov. 10, 2008. As it is very difficult to get the brain tissue samples from normal people, the control samples are all from the adjacent normal tissues of glioblastomas patients. Thus we focus on detecting the effect of somatic difference on disease, which is also a common approach in many other cancer studies. We used 254 samples for gene expression and pathway analysis, 250 samples for microRNA expression analysis, 247 samples common in microRNA and gene expression datasets for miRNA targets analysis.

### Gene expression analysis

A total of 1,236 genes were identified to be significantly differentially expressed between tumour and normal tissues. The results were given in Additional file 1. To further investigate the function of these differentially expressed genes, we used DAVID [8,9], bioinformatics resources and pathway analysis [10] for systematic and integrative analysis of large gene lists. 1,221 of 1,236 differentially expressed genes had annotations in DAVID Functional Annotation Tools. We carried out gene set enrichment analysis to indentify the most enriched gene function annotation terms (GO terms) [11] in the list of 1,221 annotated differentially expressed genes. (See methods for details.) The top ten enriched GO terms in the list of differentially expressed genes were shown in Table 1, suggesting these genes were enriched in brain and mainly associated with Nervous system development and function. The detailed information, for example, genes which shared the GO terms was given in Additional file 1.

**Table 1:** The top ten GO terms most enriched in the differentially expressed gene list

*Category*	*Term*	*Annotation*	*Count*	*PValue*
Cellular Component	GO:0045202	synapse	57	1.39E-15
Biological Process	GO:0006810	transport	288	3.10E-14
Biological Process	GO:0007268	synaptic transmission	69	8.42E-14
Biological Process	GO:0019226	transmission of nerve impulse	75	1.05E-13
Biological Process	GO:0051234	establishment of localization	292	6.89E-13
Cellular Component	GO:0043005	neuron projection	34	8.64E-13
Biological Process	GO:0051179	localization	326	2.36E-12
Cellular Component	GO:0016020	membrane	548	8.36E-10
Molecular Function	GO:0015075	ion transmembrane transporter activity	100	1.98E-09
Biological Process	GO:0007269	neurotransmitter secretion	19	2.62E-09

DAVID also could cluster similar functional GO terms together. The first two enriched GO term groups in the differentially expressed gene list were all the function terms relevant to brain and neuron. They were: 1) GOTERM Cellular Component including five terms: neuron projection, cell projection, dendrite, cell soma, and axon. 53 genes belong to this cluster including CDK5 , SNCG , UCHL1 , FREQ.

According to NCBI Entrez gene annotation [12], it was reported that the deregulation of gene CDK5 causes neuronal death and neurodegenerative diseases. Gene SNCG encodes a member of the synuclein family of proteins which are believed to be involved in the pathogenesis of neurodegenerative diseases. Mutations in this gene have also been associated with breast tumor development. Gene UCHL1 is specifically expressed in the neurons and in cells of the diffuse neuroendocrine system. Mutations in this gene may be associated with Parkinson disease. FREQ gene encodes calcium-binding proteins expressed predominantly in neurons. The protein encoded by this gene is associated with secretory granules and modulates synaptic transmission and synaptic plasticity. 2) GOTERM Biological Process including twenty one terms: synaptic transmission; transmission of nerve impulse; neurotransmitter secretion; regulated secretory pathway; generation of a signal involved in cell-cell signaling; regulation of neurotransmitter levels; neurological system process; cell-cell signaling; exocytosis; SNARE binding; secretory pathway and so on. A total of 336 genes belong to this cluster. The detailed information for this two GO term groups were given in Additional file 1.

### Pathway analysis

**Figure 1:**
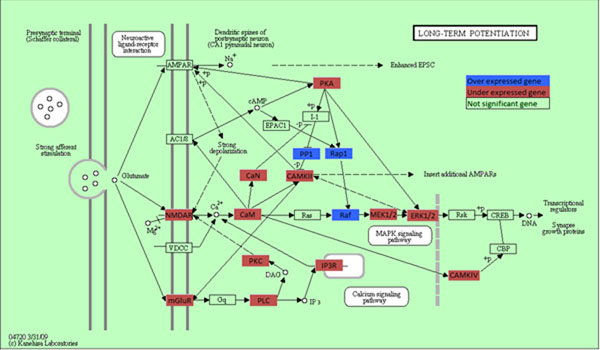
**Long term potentiation pathway** Figure 1 shows all the genes in this pathway. Significant over expressed genes are highlighted in blue and under expressed genes in red.

We first used algorithm proposed in TAPPA (Topological Analysis of Pathway Phenotype Association) [10] for pathway analysis. The results revealed that 131 pathways were significantly associated with glioblastoma (Additional file 2). The 131 associated pathways belonged to 33 functional groups, among which Cell Signaling, Neuroscience, Immunology and Expression were the most enriched pathway groups. Glioma pathway was the only significant pathway in the cancer functional group with P-value= 5.75 × 10^–7^. Similar to the GO terms enrichment analysis, we used DAVID Functional Annotation Tools to indentify which pathways were most enriched in the list of differentially expressed genes. The 40 significant pathways were also given in Additional file 2. Cell Signaling, Signal Transduction, Apoptosis and Neuroscience were the most enriched pathway groups. A total of 30 significant pathways found by both methods were shown in Additional file 2. The detailed genes information involved in the over-represented pathways was also provided. Long-term potentiation(a Nervous System pathway) and Calcium signaling pathway(a Signal Transduction pathway), were the most significantly enriched pathways with p-value *2.62* × *10*^–^*^8^* and *3.26* × *10*^–^*^8^*, respectively. There were 11significant Cell Signaling pathways, 4 significant Apoptosis pathways, 4 significant Signal Transduction pathways, 3 significant Immunology pathways, 3 significant Neuroscience pathways and 2 significant Nervous System pathways, (Some pathways may belong to different functional groups). The results suggested that the differentially expressed genes were most involved in signal, apoptosis and neuroscience pathways. Take long-term potentiation pathway as an example, Figure 1 show all the genes in this pathway, Hippocampal long-term potentiation (LTP) is a long-lasting increase in synaptic efficacy, is the molecular basis for learning and memory. 3 of the 71 genes in this pathway were significant over expressed genes and were highlighted in blue and 21 were under expressed and were highlighted in red. (One box in the figure may denote several genes)

### Analysis of differential expression of microRNA

A total of 97 microRNAs were significantly differentially expressed between tumor and normal tissues (**Additional files 3)**. To examine whether these miRNAs were associated with glioblastoma, we used miR2Disease [13] to validate our results (Updated Date: Dec. 19, 2008). MiR2Disease provides a comprehensive literature reported resource of miRNA deregulation in various human diseases. From the data in miR2Disease, 81 of the 97 significant miRNAs have been reported to associate with 84 diseases, among them, 72 miRNAs are associated with 59 cancers and brain diseases. 22 of those miRNAs have been reported to induce glioblastoma/ glioblastoma multiforme(GBM)/neuroblastoma (NB) and the expression pattern of miRNA(up-regulated or down-regulated) in published literatures is exactly the same as that in our data. Table 2 gave the p-value, expression pattern, disease and references for the 22 miRNAs. We inferred that the other 50 miRNAs which were related to other cancers and brain diseases may also be important for carcinogenesis in brain. However, further experiment validations were required to confirm our results. Among the 97 significant miRNAs, 30 miRNAs were up-regulated and 67 were down-regulated.

**Table 2:** 22 MicroRNAs related to glioblastoma/ GBM/ Neuroblastoma

*miRNA*	*P-value*	*AvgTumor*	*AvgNorm*	*Exp*	*Disease*	*Ref.*
hsa-mir-21	3.08E-24	-0.253536	-4.15408	up	GBM	[14,15,16]
hsa-mir-23a	1.45E-16	0.0400785	-1.75999	up	GBM	[14]
hsa-mir-93	5.54E-12	0.0928069	-1.32487	up	NB	[17]
hsa-mir-25	1.85E-11	-0.278892	-1.7816	up	GBM	[14]
hsa-mir-155	1.40E-10	0.2248781	-1.32519	up	GBM	[16]
hsa-mir-92	2.52E-08	-0.089368	-1.30258	up	NB	[17]
hsa-mir-210	1.74E-07	-0.203768	-1.89154	up	GBM	[16]
hsa-mir-130a	3.84E-07	-0.024653	-0.83686	up	GBM	[14]
hsa-mir-106a	1.38E-06	0.0641864	-1.04192	up	NB	[17]
hsa-mir-17-5p	2.03E-06	0.0488078	-0.97014	up	NB	[17,18]
hsa-mir-323	5.78E-36	0.1500566	1.345004	down	GBM	[16]
hsa-mir-137	1.86E-31	0.5176077	3.537668	down	GBM	[16]
hsa-mir-128a	5.47E-26	0.041749	2.428222	down	GBM	[16]
hsa-mir-154*	7.62E-23	0.1359234	0.978151	down	GBM	[16]
hsa-mir-153	1.06E-21	0.0969089	0.406899	down	GBM	[16]
hsa-mir-132	6.84E-21	0.0656709	1.852984	down	GBM	[16]
hsa-mir-7	2.53E-18	0.6340624	3.431225	down	GBM	[19]
hsa-mir-124a	1.76E-17	-0.399441	4.758568	down	GBM	[16]
hsa-mir-133b	1.36E-11	-0.00609	0.559222	down	GBM	[16]
hsa-mir-29b	1.42E-10	0.2074092	1.923726	down	GBM	[16]
hsa-mir-149	2.87E-08	0.1160284	1.636419	down	GBM	[16]
hsa-mir-133a	9.15E-08	0.0038872	0.398482	down	GBM	[16]

Exp means whether the expression pattern of microRNA is up-regulated or down- regulated, comparing the average expression value in tumor tissues(*AvgTumor*) and average expression value in normal tissues(*AvgNorm*). GBM: Glioblastoma multiforme, NB: neuroblastoma.

To further examine the function of those significant miRNAs, we need to find the target gene of miRNAs associated with glioblastomas. So we carried out the regression analysis for miRNA and gene expression.

### The regulation of gene expression by microRNA

miRNA has been thought to promote degradation of target mRNA or suppress translation of corresponding protein by matching with mRNA in the 3’-UTR region[20-23]. There is no doubt that miRNAs perform various biological functions through regulation of gene expression. To reveal the mechanisms of how miRNA regulates gene expression in GBM, we identified target genes of miRNAs and constructed miRNA target networks. Since miRNAs repress the expression of its target gene, the first step was to test the inverse relationship between the expression profile of miRNA and that of its potential targets. To achieve this, we regressed the expression of target mRNA on the expression of miRNAs and select mRNA with significant negative regression coefficients as miRNA targets. P-value for declaring significant evidence of miRNA target was *1.00* × *10*^–^*^4^*. The second step was to conduct sequence analysis which used sequence complementarities of miRNA and its target site to predict potential miRNA target genes. To achieve this, we use experimentally verified and predicted miRNA targets data from three miRNAs databases: miR2Disease[13], TarBase [24] and miRBase[25]. MiR2Disease (updated on Dec.19, 2008) and TarBase (updated on June, 2008) provided experimentally verified microRNA target genes. MiRBase predicted the target gene of miRNA by miRanda algorithm [26], where the predicted target genes and miRNAs could be downloaded directly (updated on: Oct.31, 2007).

We compiled 1,236 differentially expressed mRNAs and 97 differentially expressed miRNAs data in 237 tumor tissue sample and 10 normal tissue samples. We found two experimentally confirmed results. The literature reported that the in nasopharyngeal carcinomas underexpressed hsa-mir-29c (expression fold change (tumor/normal)=0.20) target overexpressed gene COL4A1(expression fold change(tumor/normal)=5.24) [27]. In our result, down-regulated hsa-mir-29c (differentially expressed P-value <*5.11* × *10*^–^*^12^*) targets over-expressed gene COL4A1 (differentially expressed P-value <*3.58* × *10*^–^*^6^*) with regression *β* = –*389.02* and P= *1.35* × *10*^–^*^8^*. We conclude that hsa-mir-29c is also an important miRNA in glioblastomas. Another experiment validated targets gene was LDOC1 targeted by has-miR-155[28]. The known oncogenic miRNA hsa-miR-155 can regulate a set of target genes including LDOC1, a regulator of apoptosis [29]. Our results showed that hsa-miR-155 was over-expressed (differentially expressed P-value <*1.40* × *10*^–^*^10^*) and targets under-expressed gene LDOC1 (differentially expressed P-value <*1.085* × *10*^–^*^31^*) with regression *β =* –*196.77* and P= *4.00* × *10*^–^*^15^*. We inferred that hsa-mir-155 could induce cancer through regulation of apoptosis gene LDOC1 in glioblastomas.

**Figure 2:**
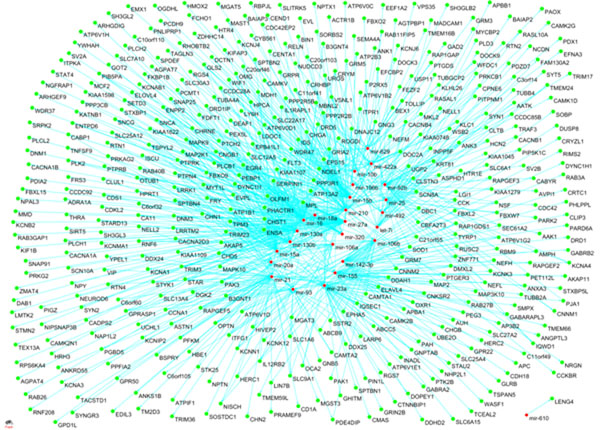
**Up-regulated miRNA and their targets** Figure 2 show the network of 26 up-regulated miRNAs and 459 under-expressed target genes.

**Figure 3:**
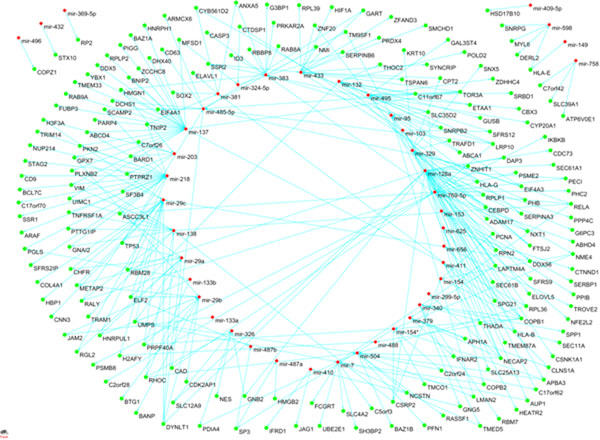
**Down-regulated miRNA and their targets** Figure 3 shows the network of 44 down-regulated miRNAs and 202 over-expressed target genes.

For predicted targets in miRBase, we found 1,094 matched miRNA-gene pairs including 70 miRNAs and 661 genes (Additional file 4). 44 down-regulated miRNAs target 202 overexpressed genes while 26 up-regulated microRNAs target 459 underexpressed genes. The up and down-regulated miRNA-gene pairs were shown in Figure 2 and Figure 3.

The 661 target genes were a subset of the 1236 significant differentially expressed genes. We examined which pathways were these genes enriched in and compared them with the previous results. 11 pathways were significant by fisher exact test in DAVID, 8 of which were the same as the pathways identified from the previous sections: Epithelial cell signaling in Helicobacter pylori infection,Cholera - Infection, Long-term potentiation, Calcium signaling pathway, Neurodegenerative Diseases, Long-term depression, Gap junction, Neuroactive ligand-receptor interaction. Three new enriched pathways include Amyotrophic lateral sclerosis (ALS), Alzheimer’s disease, Wnt signaling pathway. These differentially expressed genes were also most involved in signal and neuroscience pathways.

To investigate the function of the 661 target genes, we searched the TSGDB [30] (a tumor suppressor gene database) and DNA-Tumor Suppressor and Oncogene Database [31] and we found eight tumor suppressor genes APC, TP53, BIN1, BTG1, CDK2AP1, LDOC1, RASSF1, WFDC1 and three oncogenes: MCF2, MPL, THRA.

According to NCBI Entrez gene annotation [12], APC encodes a tumor suppressor protein that acts as an antagonist of the Wnt signaling pathway. It is also involved in other processes including cell migration and adhesion, transcriptional activation, and apoptosis. TP53 encodes tumor protein p53, which responds to diverse cellular stresses to regulate target genes that induce cell cycle arrest, apoptosis, senescence, DNA repair, or changes in metabolism. BIN1 encodes several isoforms of a nucleocytoplasmic adaptor protein, one of which was initially identified as a MYC-interacting protein with features of a tumor suppressor. Isoforms that are expressed in the central nervous system may be involved in synaptic vesicle endocytosis and may interact with dynanim, synaptojanin, endophilin, and clathrin. LDOC1 is thought to regulate the transcriptional response mediated by the nuclear factor kappa B (NF-kappaB). The gene has been proposed as a tumor suppressor gene whose protein product may have an important role in the development and/or progression of some cancers. RASSF1 encoded protein was found to interact with DNA repair protein XPA. The protein was also shown to inhibit the accumulation of cyclin D1, and thus induce cell cycle arrest. WFDC1 gene is mapped to chromosome 16q24, an area of frequent loss of heterozygosity in many cancers. Owing to its location and a possible growth inhibitory property of its gene product, this gene is suggested to be a tumor suppressor gene. MCF2 is a member of a large family of GDP-GTP exchange factors that modulate the activity of small GTPases of the Rho family. Five-prime recombinations result in the loss of N-terminal codons, producing MCF2 variants with oncogenic potential.

**Figure 4:**
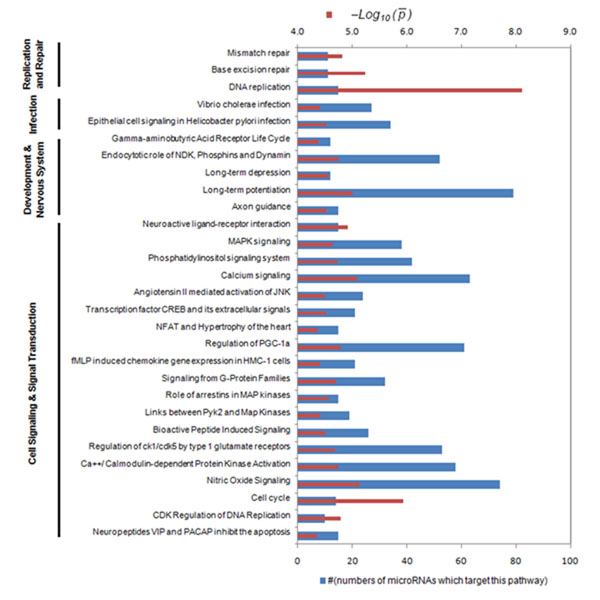
**Down-regulated miRNA and their targets** A total of 29 pathways which were targeted by more than 10 miRNAs were shown in Figure 4. The blue bar indicated that the pathway was targeted by the number of miRNAs. The red bar gave the negative logarithm to the base 10 of average P-value indicating the significance of enrichment of the pathway in miRNA targets.

To further investigate the function of target genes, we identified the miRNA targeted pathways by right-tail fisher exact test, which tested enrichment of pathways in the miRNA target gene set. A total of 83 pathways targeted by 94 miRNAs were listed in Additional file 5 after Bonferroni correction for multiple tests (p-values<*1.00* × *10*^–^*^4^*). Many of the pathways were targeted by more than one miRNA. We shown 29 pathways which were targeted by more than 10 miRNAs in Figure 4. Long-term potentiation (Nervous System pathway) was targeted by 79 (the most) miRNAs and Nitric Oxide Signalling (Signalling pathway) was targeted by 74 (the second most) miRNAs. We can see that the differentially expressed miRNAs most frequently targeted genes in Cell Signalling and Nervous System. The red bar gave the negative logarithm with 10 base of average p-value indicating the significance of enrichment of the pathway in the miRNA targets. The DNA replication pathway and the cell cycle pathway have the smallest average P-value *7.70* × *10*^–^*^9^* and the second smallest P- value *1.17* × *10*^–^*^6^*. P-values of Long-term potentiation pathway (*7.29* × *10*^–^*^6^*) and Nitric Oxide Signalling pathway (*9.99* × *10*^–^*^6^*) were also small.

## Conclusions

In this paper, we performed detailed analysis of differential expression of gene and miRNA between tumor tissues and normal brain tissues in glioblastomas. We also performed gene sets enrichment analysis to find the enriched GO terms and pathways. Most of the genes were enriched in Nervous system associated GO terms and Cell Signaling and Neuroscience associated pathways. 22 differentially expressed miRNAs were related to Glioblastoma multiforme or neuroblastoma. To study the regulation of gene expression by miRNA, we combined the sequence predicted miRNA targets in miRBase database, experiment validated miRNA targets in TarBase and miR2Disease database with our predictions from the gene and miRNA expression profiles and found 2 experiment validated targets and 1,094 predicted targets. Further function analysis of target genes suggests that miRNAs most frequently targeted genes in Cell Signalling and Nervous System. However, the number of normal tissues in the studies is small. More samples are needed for further investigation.

## Methods

### DAVID bioinformatics resources

The **D**atabase for **A**nnotation, **V**isualization and **I**ntegrated **D**iscovery **(DAVID)** provides a comprehensive set of functional annotation tools for investigators to understand biological meaning behind large list of genes [8,9] (http://david.abcc.ncifcrf.gov/). After inputting large gene lists, it automatically calculates and identifies enriched biological themes, particularly GO terms and pathways; discovers enriched functional-related gene groups and clusters redundant annotation terms. For any one GO terms, right tail modified Fisher Exact was used to determine whether the number of genes with this GO terms is enriched in the differentially expressed gene list compared to the number of genes with this GO terms in all the 19,439 genes on HG-U133A array(Background). For any one pathway, right tail modified Fisher Exact was used to determine whether the number of genes within this pathway is enriched in the differentially expressed gene list compared to the number of genes within all KEGG or Biocarta pathways. The smaller the p-value was, much more enriched in the GO terms or pathway than by random chance.

### Pathway-based differential expression analysis

We used algorithm proposed in TAPPA (Topological Analysis of Pathway Phenotype Association) [10] for pathway analysis. It calculated a Pathway Connectivity Index for each pathway and then evaluates its correlation to the phenotype variation. Gene connections of 162 KEGG pathways with gene number higher than 8 were collected in that paper and used for PCI calculation. For those pathways with no edge connections collected, PCI would degenerate into the average of gene expression values. Totally 501 pathways from KEGG [32] and Biocarta [33] were assembled in our analysis. The p-value for declaring significance after Bonferroni correction for multiple tests was 1 × 10^–4^.

### Statistical analysis

The differential expression of the gene and microRNA were tested by *T*-test and Mann-Whitney Test. The thresholds for declaring significance after Bonferroni correction for multiple tests were 4.15 × 10^–6^ and 9.36 × 10^–5^, for gene and miRNA respectively. Linear regression was used to investigate the relationships between miRNA and gene expressions. The linear model took its common form: where y is an n-by-1 vector of observations, such as gene expression. X is an n-by-p matrix of regressors, such as miRNA expression, β is a p-by-1 vector of parameters; known as regression coefficient and ε is an n-by-1 vector of random disturbances. Right-tail fisher exact test were used to test for the enriched Gene Ontology Terms, pathways in the datasets. Matlab code for T-Test, Mann-Whitney Test and linear regression was attached in Additional files 6.

## Competing interests

The authors declare that they have no competing interests.

## Authors’ contributions

HD was responsible for acquisition, analysis and interpretation of data and wrote the draft. HS, LL, XF did the statistical analysis and the programming. LJ and MX provided the administrative, technical and material support. MX participated in its design and coordination and helped to draft the manuscript. All authors read and approved the final manuscript.

## Supplementary Material

Additional file 1 A total of 1236 genes were significantly differentially expressed by both T-test and Mann-Whitney Test after Bonferroni correction for multiple tests. The top 10 enriched GO terms and the first 2 significant GO terms clusters were given in sheet 2 and sheet 3, respectively. Click here for file

Additional file 2 Three sheets gave the list of 131 significant pathways indentified by TAPPA method, the list of 40 significant pathways indentified by Fisher Exact Test and the list of 30 pathways shared by two methods, respectively. Click here for file

Additional file 3 A total of 97 microRNAs were significantly differentially expressed by both T-test and Mann-Whitney Test after Bonferroni correction for multiple tests. Click here for file

Additional file 4 The 1,094 matched miRNA-gene pairs including 70 microRNAs and 661 genes predicted by both our results and miRanda algorithm. Click here for file

Additional file 5 A total of 83 pathways targeted by 94 microRNAs were listed after Bonferroni correction for multiple tests. Click here for file

Additional files 6 We have attached the Matlab code for T-Test, Mann-Whitney Test and linear regression in additional files 6 Click here for file
